# Chemotherapy cardiotoxicity research in cancer patients: a bibliometric and visual analysis (1994–2024)

**DOI:** 10.3389/fonc.2025.1502361

**Published:** 2025-05-15

**Authors:** Guoming Chen, Huiping Zhou, Chengbin Wang, Rui Qin, Qingyi Yang, Yingyue Hou, Meizhen Zhang, Cheng Zhang, Ning Wang, Yibin Feng

**Affiliations:** ^1^ School of Chinese Medicine, Li Ka Shing Faculty of Medicine, The University of Hong Kong, Hong Kong, Hong Kong SAR, China; ^2^ Foshan Clinical Medical College, Guangzhou University of Chinese Medicine, Foshan, China; ^3^ Shenzhen Clinical Medical College, Guangzhou University of Chinese Medicine, Shenzhen, China; ^4^ First Clinical Medical College, Guangzhou University of Chinese Medicine, Guangzhou, China; ^5^ Clinical Medical College of Acupuncture Moxibustion and Rehabilitation, Guangzhou University of Chinese Medicine, Guangzhou, China; ^6^ School of Information Engineering, Guizhou University of Traditional Chinese Medicine, Guizhou, China

**Keywords:** bibliometric analysis, cancer, chemotherapy, cardiotoxicity, CiteSpace, VOSviewer

## Abstract

**Background:**

Recent advancements in medical technology have significantly boosted the survival rates of cancer patients. The potential toxicities that cancer treatments may induce in other areas of the human body have garnered increasing attention. Chemotherapy, a pivotal component of anticancer therapy, heightens the risk of cardiac damage and contributes to various cardiovascular complications. To comprehensively assess the research landscape concerning chemotherapy-induced cardiotoxicity in cancer patients, this study conducted a visual analysis of pertinent articles utilizing bibliometric tools.

**Method:**

We used CiteSpace, VOSviewer, to analyze the temporal and spatial distribution, disciplinary categories, authorship, references, subject terms, and keywords of 4460 articles retrieved from the Web of Science core collection from 1994 to January 21, 2024.

**Results:**

Contributions to this field emanated from 100 countries/regions and 4,343 research institutions, with the United States, China, and Italy emerging as the most prolific contributors in terms of publication volume. The Journal of Clinical Oncology emerged as the primary journal in terms of publications and co-citations within this domain, with Bonnie Ky identified as the most prolific author. Common keywords included breast cancer, doxorubicin, heart failure, trial, adjuvant chemotherapy, paclitaxel, and risk. Terms such as echocardiography, nanoparticles, mechanisms, prevention, society, oxidative stress, inflammation, consensus, and global longitudinal strain delineated the current research frontier in this field. Additionally, the study provided a detailed examination of the fundamental aspects of research in this domain. Presently, key research focuses in this area include elucidating the mechanisms of chemotherapy-induced cardiotoxicity, monitoring and evaluating such cardiotoxicity, and developing strategies for its prevention and treatment.

**Conclusion:**

This study provides a comprehensive overview of chemotherapy-induced cardiotoxicity research from 1994 to 2024. Future research should prioritize personalized risk assessment and interdisciplinary collaboration to meet the clinical needs of the cardiovascular oncology field.

## Introduction

Cancer accounts for one out of every seven fatalities globally. As substantiated by a recently released report from the World Health Organization, given the escalating geriatric populace, the international cancer load is anticipated to ascend to 21.7 million new cases and 13 million mortalities by the year 2030 (1). It results in human disability and mortality, increasingly emerging as a significant health concern and a burden for the majority of global public healthcare systems (2). The World Health Organization’s data projects that there will be approximately 27 million cancer cases by 2050, with an annual death toll of 17.5 million (3). China has emerged as the leading nation in cancer, with data from the National Cancer Center in February 2022 indicating that there were 4.064 million new cases and 2.414 million deaths from cancer in the country in 2016. Both figures have been on the rise, leading to annual medical expenses exceeding 220 billion yuan (4). At present, cancer treatment adopts various strategies in clinical practice, including surgery, radiotherapy, chemotherapy, immunotherapy, and laser therapy (5). Over the past few decades, molecular targeted therapy and immunotherapy have shown efficacy in a subgroup of cancer patients (6). Advancements in sequencing technology have led researchers to recognize cancer as a complex, hereditary condition (7). Many of these mutated genes have the potential to cause cancer, and ongoing research is exploring treatment methods for these gene mutations. Immunotherapy has been validated and shown promising results in numerous cancer types, yet this therapy still faces challenges (8). Despite these advances, chemotherapy remains a viable option for cancer treatment at present (9). The primary objective of chemotherapy is to establish the fundamental principle of selectively eliminating malignant cells without causing significant harm to normal cells. Nevertheless, healthy cells are still eliminated, resulting in significant adverse effects such as bone marrow suppression, hair loss, and gastrointestinal issues (10). Some studies have found that the incidence rate of subclinical cardiac toxicity caused by chemotherapy is three times as high as that of clinical cardiac toxicity, which deserves more attention (11). Individuals who have survived malignant tumors over the long term may ultimately succumb to cardiovascular disease rather than the original malignancy (12). Cardiovascular disease has emerged as a primary cause of non-cancer mortality among cancer patients (13). Chemotherapy, as a primary treatment modality for cancer, can elevate the risk of heart injury, ultimately leading to various early and late cardiovascular conditions, including heart failure, arrhythmia, cardiomyopathy, and more. Consequently, it is imperative to monitor for cardiotoxicity during the chemotherapy process for patients (14).

In previous studies, significant progress has been made in understanding the mechanisms and preventing cardiac toxicity caused by chemotherapy, yet a comprehensive summary of research content and progress remains lacking. Bibliometric analysis is a scientifically quantitative method of literature review that aids in identifying research trends, discovering emerging fields and collaborations, and supplying information for strategic planning and resource allocation in research (15). At present, bibliometric analysis mainly utilizes software such as CiteSpace and VOSviewer (16). It offers researchers a comprehensive grasp of fundamental data and dynamic trends, facilitates an understanding of knowledge structures, and enables exploration of developmental trends in relevant fields, which aids researchers in formulating guidelines, identifying research hotspots, and evaluating research trends (17, 18).

Since chemotherapy technology is a common and crucial treatment for cancer patients, with numerous successful clinical applications and ongoing development, the cardiac toxicity caused by its therapy is becoming increasingly apparent. Therefore, understanding and preventing chemotherapy-induced cardiac toxicity in cancer patients are essential tasks in clinical medicine and nursing. By conducting bibliometric research on this topic, we aim to comprehend the safety and toxic side effects of chemotherapy techniques, and inform clinical practice and refine evidence-based strategies for the further development of chemotherapy technology applications. In this study, based on the Science Citation Index Expanded (SCIE) database of the Web of Science core collection, bibliometric statistics and analysis were conducted on the research literature on chemotherapy-induced cardiac toxicity in cancer patients published since the database was established. The output, hotspots, frontiers, and trends of chemotherapy research in this patient population were revealed from the aspects of literature output and influence. This provides scientific guidance for the prognosis or prevention and treatment of cardiac toxicity in patients after chemotherapy.

## Materials and methods

### Data acquisition

Web of Science is a widely used and recognized academic database encompassing over 12,000 scholarly journals. Many researchers and academic institutions rely on it for literature retrieval and citation analysis (19). To retrieve relevant articles, a search will be conducted in the Science Citation Index Expanded (SCIE) database, which is part of the Web of Science Core Collection (WoSCC). The search will cover the period from the database’s inception in 1994 until January 21, 2024. Using a search strategy agreed upon by all authors, the following criteria were employed: (((((((((((((((((TS=(Neoplasms)) OR TS=(Neoplasm)) OR TS=(Neoplasm, Benign) OR TS=(Tumors)) OR TS=(Cancer)) OR TS=(Malignancy)) OR TS=(Neoplasia)) OR TS=(Cancers)) OR TS=(Neoplasms, Malignant)) OR TS=(Neoplasias)) OR TS=(Malignancies)) OR TS=(Malignant Neoplasms)) OR TS=(Neoplasm, Malignant)) OR TS=(Malignant Neoplasm)) OR TS=(Benign Neoplasms)) OR TS=(Benign Neoplasm)) OR TS=(Neoplasms, Benign)) OR TS=(Tumor)) AND TS=(Chemotherapy)) OR TS=(Chemotherapies)) AND ((((TS=(Cardiotoxicities)) OR TS=(cardiotoxicity)) OR TS=(Cardiac Toxicity)) OR TS=(Cardiac Toxicities)) OR TS=(Toxicity, Cardiac). In order to facilitate further analysis of the literature content, the articles were restricted to the English language and the research article type. Then, complete records and references cited were extracted from relevant publications. They were saved in plain text format for further research purposes.

### Data analysis

**Figure 1:**
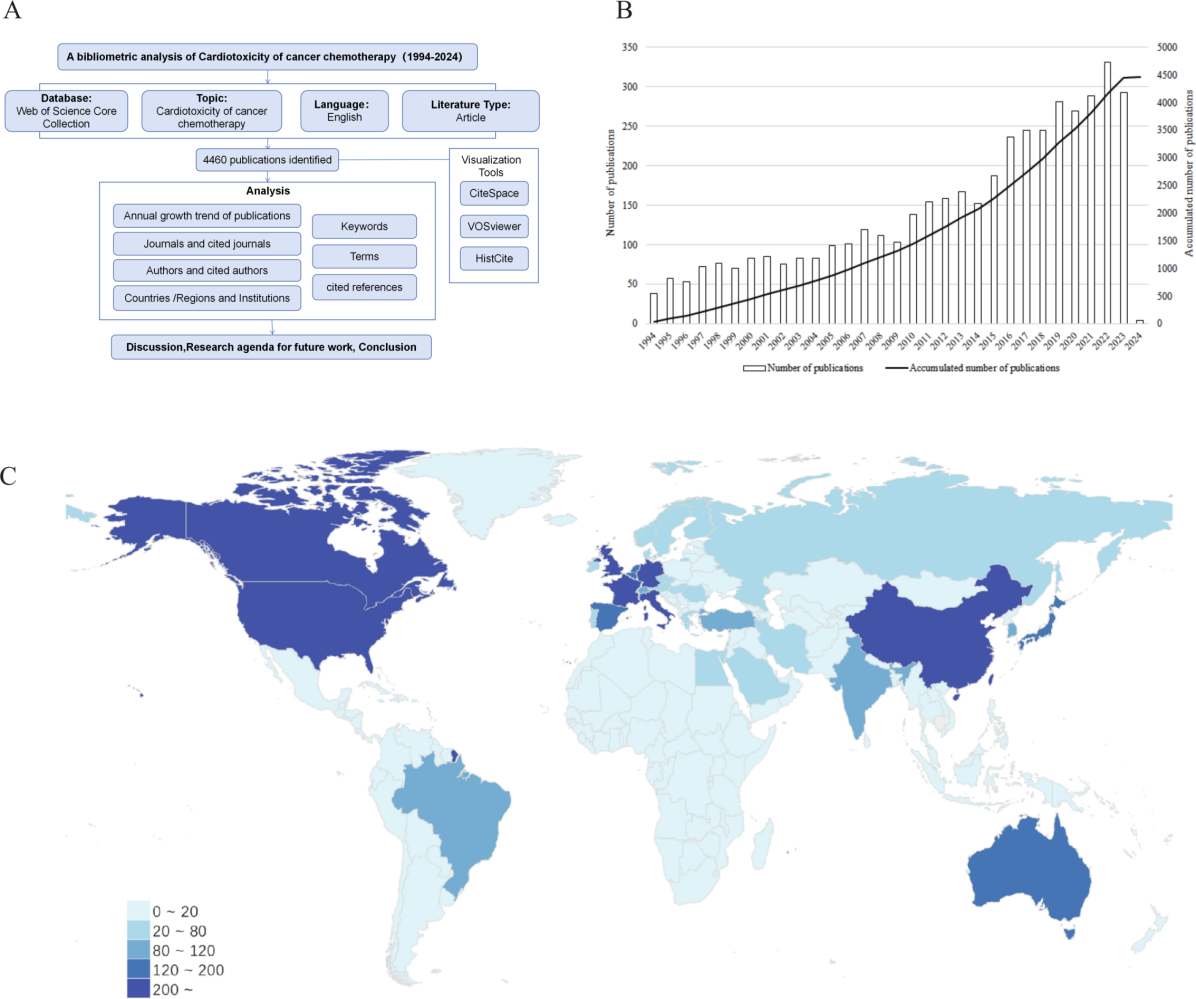
**(A)** Flowchart depicting the article selection process. **(B)** Annual publication growth bar chart and annual cumulative publication line chart of cancer chemotherapy cardiotoxicity. Annual publication refers to the number of articles published each year, and the cumulative annual publication is the number of articles published up to that year. **(C)** Comparative map of the cumulative number of papers published in each country. The darker the color, the more posts there are.

This study employed three bibliometric tools to assist in our analysis, namely VOSviewer (version 1.6.19), CiteSpace (version 6.2.R4 Advanced), and HistCite (version Pro 2.1). In this research, VOSviewer was utilized to visualize collaboration networks of institutions, countries, authors, and terms. CiteSpace was employed for visualizing keyword co-occurrence, keyword clustering, keyword time zone, keyword burst analysis, cited journals, and cited references. It is important to note that due to the cumulative nature of thresholds, some recent keywords may not have reached a count of 30. Therefore, this study included the top 3 high-frequency keywords for each year from 2016 to 2024 to supplement the keyword time zone analysis. HistCite was solely used for analyzing journal publication counts. (**Figure 1A**)

## Results

### Annual growth trend of publications

Through a search of the WOS database, a total of 4,460 studies on chemotherapy-induced cardiac toxicity in cancer patients were identified within the time frame since its establishment. **Figure 1B** displays the distribution of annual publications on chemotherapy-induced cardiotoxicity in cancer patients, with the highest number of publications in 2022, amounting to 331. By analyzing the publication volume and literature trend, we can comprehend the level of development in this field during a specific period. Despite fluctuations since 1994, the publication volume has continued to increase. This trend signifies that research on cardiac toxicity caused by tumor chemotherapy has garnered increasing attention in recent years.

### Institutions/regions and countries analysis

**Figure 2:**
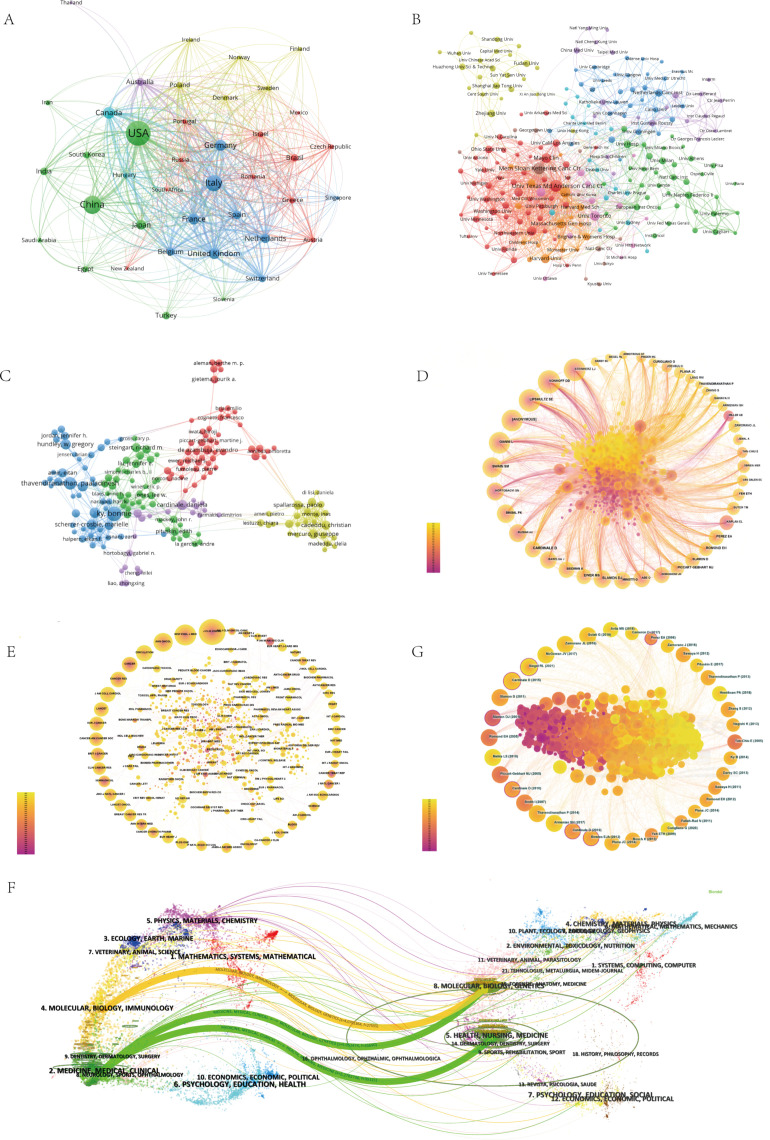
**(A)** This network diagram was created by the software VOSviewer based on all articles. This network diagram selects countries with a frequency greater than 15. The more frequently these countries appear, the larger the circle, and the darker the color of the circle. The line connecting two circles can represent the degree of connection between these two countries, and the higher the correlation, the thicker the line. **(B)** This network diagram selects institutions with a frequency greater than 10. The more frequently these institutions appear, the larger the circle and the darker the color of the circle. The line connecting two circles can represent the degree of connection between these two institutions, and the higher the correlation, the thicker the line. **(C)** Network diagram of the author’s published articles on cancer chemotherapy-induced cardiac toxicity. The larger the node, the more articles are published. The node connection line represents the strength of the relationship between authors. **(D)** Network diagram of co-cited authors involved in the cardiac toxicity of cancer chemotherapy. Each node represents an author. The size of nodes is positively correlated with the number of citations by authors, and the connection between two nodes represents the collaboration between two authors on the same article. **(E)** CiteSpace network visualization of journals related to cardiotoxicity of chemotherapy. Nodes represent journals and lines representing co-occurrence between journals connect them. The node size is proportional to the frequency of the journals. The outermost layer of a node with a purple circle indicates the high centrality of the represented keyword. **(F)** The dual-overlay of journals related to the cardiotoxicity of chemotherapy. The citation graph is displayed on the left side, while the cited graph is shown on the right side. The curve depicts the verification line, with various reference relationships displayed in distinct colors. **(G)** Network diagram of co-cited literature related to cardiac toxicity of cancer chemotherapy. Each node signifies a reference, with node size positively correlated to citation frequency. The linkage between two nodes indicates references cited within the same article.

**Figure 1C** provides a global view of the quantity of published articles in this domain. **Figure 2A** shows a network map created with VOSviewer software, displaying publications on cardiotoxicity in cancer chemotherapy across countries, as indexed in the Web of Science (WoS) database till the present. **Figure 2A** selects countries with a frequency greater than 15. The United States, China, Italy, Canada, Germany, the United Kingdom, France, Japan, the Netherlands, and Spain rank among the top ten countries in terms of the quantity of published articles. The United States leads with 1,391 publications, accounting for 31.19% of the total 4,460 articles, followed by China with 686 (15.38%), Italy with 544 (12.20%), Canada with 285 (6.39%), and Germany with 253 (5.67%). The United States and the United Kingdom exhibit the strongest collaborative efforts with other countries. Among all countries, the ones most closely connected to the United States, which has the highest publication output, are China, Italy, and Germany, with respective collaborative strengths of 9.64%, 7.11%, and 6.47%.

**Figure 2B** shows a network map of institutional cooperation, revealing the strength of their interconnections. The top five institutions in terms of publication output are the MD Anderson Cancer Center in Texas, USA, the Memorial Sloan Kettering Cancer Center, a private cancer center in the USA, Dana Farber Cancer Institute, an affiliate of Harvard Medical School specializing in cancer, the University of Toronto, and the Mayo Clinic, all located in the United States and Canada, which are among the top five countries for publication volume. Compared to the number one ranked institution, MD Anderson Cancer Center, the Dana Farber Cancer Institute affiliated with Harvard Medical School has a stronger collaboration power with other institutions, with a total collaboration strength of 266, accounting for 6.27% of the total inter-institutional collaboration strength of 4243, while MD Anderson comprises just 5.84%. The University of Toronto in Canada’s collaboration strength with other countries stands at 138, representing 3.25% of the total collaboration strength.

### Authors and co-cited authors analysis

According to the visualization analysis by VOSviewer authors (**Figure 2C**), a total of 28815 authors participated in the study of cardiac toxicity in cancer chemotherapy therapy. In the context of article publication volume, Bonnie Ky (29 publications) has the highest number of publications, followed by Paaladinesh Thavendiranathan (23 publications), and Juan Carlos Plana (17 publications). Co-cited authors refer to authors who are also cited in the article, and based on bibliometric analysis, a co-author network diagram is generated. According to the visualization analysis of CiteSpace co-cited authors (**Figure 2D**), out of 900 co-cited authors, 59 were co-cited more than 100 times, 5 authors were cited more than 500 times, and one author was cited more than 600 times. The article published by the author is mainly related to cardiotoxicity, exploring the relationship between cardiotoxicity and various treatment methods for cancer patients, as well as its detection and management guidelines (20–22). The top five co-cited authors are Daniela Cardinale (680), [ANONYMOUS] (546), Dennis J Slamon (529), Steven E Lipshultz (518), and Sandra M Swain (518).

### Distribution of journals

A total of 941 academic journals have published articles on the research of chemotherapy-induced cardiotoxicity in cancer patients. **Table 1** presents the top 10 journals ranked by publication count. Among them, 6 journals are located in the first quartile (Q1) of the Journal Citation Reports (JCR). The publishers are from the United States, the United Kingdom, and Greece, with the majority (6) being from the United States. The journal’s co-occurrence analysis is illustrated in **Figure 2E**. **Table 2** displays the top 10 most cited journals. These journals are all published by publishers from the United States or the United Kingdom and are located in the Q1 area of the Journal Citation Reports (JCR). The impact of journals depends on the number of times they are collectively cited, which reflects their necessary influence on specific topics (23). Interestingly, the Journal of Clinical Oncology is the journal with the highest number of publications associated with the topic and is also the most frequently co-cited journal. The dual-map overlay of journals (**Figure 2F**) can reflect the thematic distribution of academic journals (24). As shown in **Figure 2A**, the green paths indicate that articles published in the “8. MOLECULAR/BIOLOGY/GENETICS” and “5. HEALTH/NURSING/MEDICINE” journals are consistently cited in research published in the “2. MEDICINE/MEDICAL/CLINICAL” journal. The yellow paths suggest that research published in the “4. MOLECULAR/BIOLOGY/IMMUNOLOGY” journal often cites articles from the “8. MOLECULAR/BIOLOGY/GENETICS” journal.

**Table 1:** The top 10 prolific journals associated with cardiotoxicity of chemotherapy.

Rank	Source	Article	Country	IF	H-index	JCR-c
1	JOURNAL OF CLINICAL ONCOLOGY	183	United States	45.4	494	Q1
2	ANNALS OF ONCOLOGY	108	England	50.5	210	Q1
3	BREAST CANCER RESEARCH AND TREATMENT	103	United States	3.8	139	Q2
4	CANCER	88	United States	6.2	277	Q1
5	CANCER CHEMOTHERAPY AND PHARMACOLOGY	68	United States	3	100	Q3
6	ANTICANCER RESEARCH	61	Greece	2	110	Q4
7	ONCOLOGIST	58	United States	5.8	145	Q1
8	ANTI-CANCER DRUGS	57	United States	2.3	88	Q3
9	EUROPEAN JOURNAL OF CANCER	54	England	8.4	193	Q1
10	BRITISH JOURNAL OF CANCER	53	England	8.8	212	Q1

**Table 2:** The top 10 co-cited journals related to cardiotoxicity of chemotherapy.

Rank	Co-cited journals	Citations	Country	IF	H-index	JCR-c
1	JOURNAL OF CLINICAL ONCOLOGY	3345	United States	45.4	494	Q1
2	NEW ENGLAND JOURNAL OF MEDICINE	2345	United States	158.5	933	Q1
3	CANCER	2009	United States	6.2	277	Q1
4	ANNALS OF ONCOLOGY	1874	England	50.5	210	Q1
5	CIRCULATION	1459	United States	37.8	570	Q1
6	CANCER RESEARCH	1366	United States	11.2	411	Q1
7	JOURNAL OF THE AMERICAN COLLEGE OF CARDIOLOGY	1286	United States	24.4	394	Q1
8	LANCET	1231	England	168.9	700	Q1
9	EUROPEAN JOURNAL OF CANCER	1166	England	8.4	193	Q1
10	BRITISH JOURNAL OF CANCER	1045	England	8.8	212	Q1

### Top cited research in chemotherapy cardiotoxicity

Co-cited literature analysis holds immense significance in scientific research as it provides a theoretical foundation and knowledge framework for further exploration. When two references are simultaneously cited, there may exist a correlation between their content. As the number of co-citations between these two literary works increases, the correlation between them strengthens accordingly. Therefore, it is imperative to conduct statistical analysis on co-cited literature (**Figure 2G**). Over the past 20 years, there have been 92,830 co-cited articles on the cardiotoxicity of tumor chemotherapy. The article titled “2016 ESC Position Paper on Cancer Treatments and cardiovascular toxicity developed under the guidelines of the ESC Committee for Practice: The Task Force for Cancer Treatments and cardiovascular toxicity of the European Society of Cardiology (ESC)” holds the highest number of citations, with a total of 136 citations, ranking first. This article aims to develop the optimal treatment plan for patients experiencing cardiac toxicity caused by cancer treatment, while fully considering the impact of treatment effectiveness and the balance between the risk and benefit of specific diagnostic or treatment methods (25).

### Top research topics in chemotherapy cardiotoxicity

**Figure 3:**
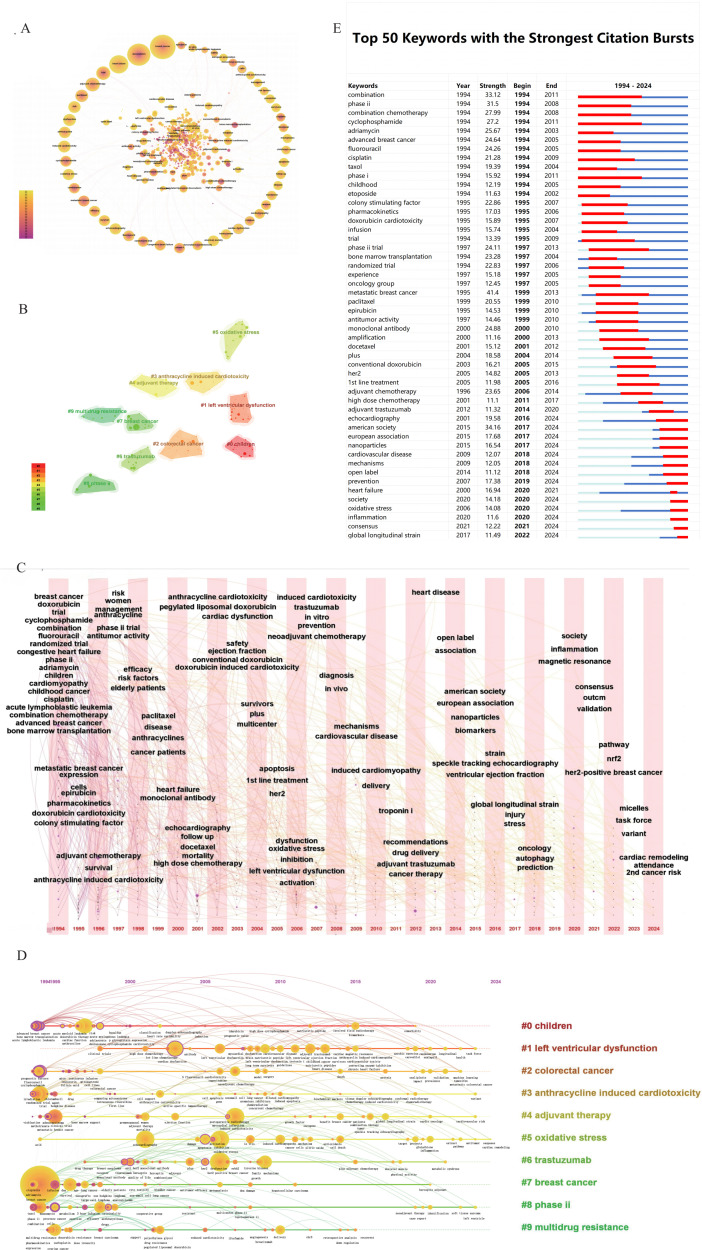
**(A)** The CiteSpace network visualization of keywords related to chemotherapy-induced cardiotoxicity. Nodes represent keywords, and lines denote co-occurrences between keywords. The nodes’ circles are arranged in a counterclockwise order based on their frequency of occurrence. The size of each node is proportional to the frequency of the corresponding keyword. The outermost layer of purple circular nodes signifies keywords with higher centrality within the network. **(B)**The cluster of keywords pertaining to chemotherapy-induced cardiotoxicity is visually represented, with each distinct color indicating a separate thematic cluster. **(C)** The keyword time zone map is associated with the cardiotoxicity of chemotherapy. Each column of keywords is arranged from top to bottom based on the frequency of occurrence, with keywords higher up indicating a higher frequency of occurrence. Clusters are distinguished by different colors, and connections between keywords signify their co-occurrence. **(D)** Timeline view of keywords related to chemotherapy-induced cardiotoxicity. The chronological representation of keywords associated with chemotherapy-induced cardiotoxicity illustrates clusters as horizontal lines, with cluster #0 being the most substantial. Subsequent clusters are depicted in decreasing size. Node sizes within each cluster correspond to their co-citation frequencies, while lines connecting nodes indicate co-citation relationships. The year of occurrence for each node signifies the initial co-citation date. **(E)** The top 50 keywords exhibiting robust citation burstiness are depicted. A blue line denotes the timeline, with bursts highlighted in red to indicate the commencement year, conclusion year, and duration of each burst.

Keywords serve as indicators of the central themes of an article. Co-occurrence analysis of keywords not only facilitates the identification of active domains within a research field but also aids in the exploration and delineation of emerging trends and focal points within the academic discourse (19). After removing irrelevant keywords and merging synonymous keywords, a total of 488 keywords were obtained from 4460 papers, with labels representing keywords that appeared 50 times or more. (**Figure 3A**) From **Figure 3A**, it can be observed that the most frequent keywords related to the cardiac toxicity of cancer chemotherapy include breast cancer, doxorubicin, heart failure, trial, adjuvant chemotherapy, paclitaxel, risk, dysfunction, anthracyline, induced cardiotoxicity, cyclophosphamide, oxidative stress, and combination. In order to capture the key themes in studies related to the cardiac toxicity of cancer chemotherapy, keyword clustering was performed, and the top ten largest clusters were identified. (**Figure 3B**) To capture the key themes of research related to cancer chemotherapy-induced cardiac toxicity, keyword clustering was performed, resulting in the identification of the top ten clusters. They are as follows: “children” (Cluster 0), “left ventricular dysfunction” (Cluster 1), “colorectal cancer” (Cluster 2), “anthracycline induced cardiotoxicity” (Cluster 3), “adjuvant therapy” (Cluster 4), “oxidative stress” (Cluster 5), “trastuzumab” (Cluster 6), “breast cancer” (Cluster 7), “phase ii” (Cluster 8), “multidrug resistance” (Cluster 9). Additionally, we generated keyword timelines (**Figure 3C**) and keyword time zone graphs(**Figure 3D**), which facilitate the visualization of the evolution of research hotspots. Keyword burst is considered another key indicator of research frontiers. **Figure 3E** illustrates the top 50 keywords with the highest citation bursts. Keywords such as “Echocardiography,” “American Society,” “European Association,” “Nanoparticles,” “Cardiovascular Disease,” “Mechanisms,” “Open Label,” “Prevention,” “Society,” “Oxidative Stress,” “Inflammation,” “Consensus,” and “Global Longitudinal Strain” have experienced sustained citation bursts until 2024 and are still ongoing. Additionally, we identified the top five chemotherapy approaches based on the number of citations, as detailed in **Table 3**. In this table, Degree centrality denotes the number of connections a node has with other nodes in a network. A higher degree in a network indicates higher centrality, suggesting greater importance of the node in the network.

**Table 3:** Top 5 chemotherapies in the publications.

Chemotherapy	Number of citations	Degree	Centrality	Burst
doxorubicin	853	4	0.03	8.34
paclitaxel	265	6	0.10	20.57
anthracycline	248	5	0.06	0.00
cyclophosphamide	237	7	0.09	27.33
fluorouracil	209	9	0.15	24.37

### Subject terms and clustering analysis

**Figure 4:**
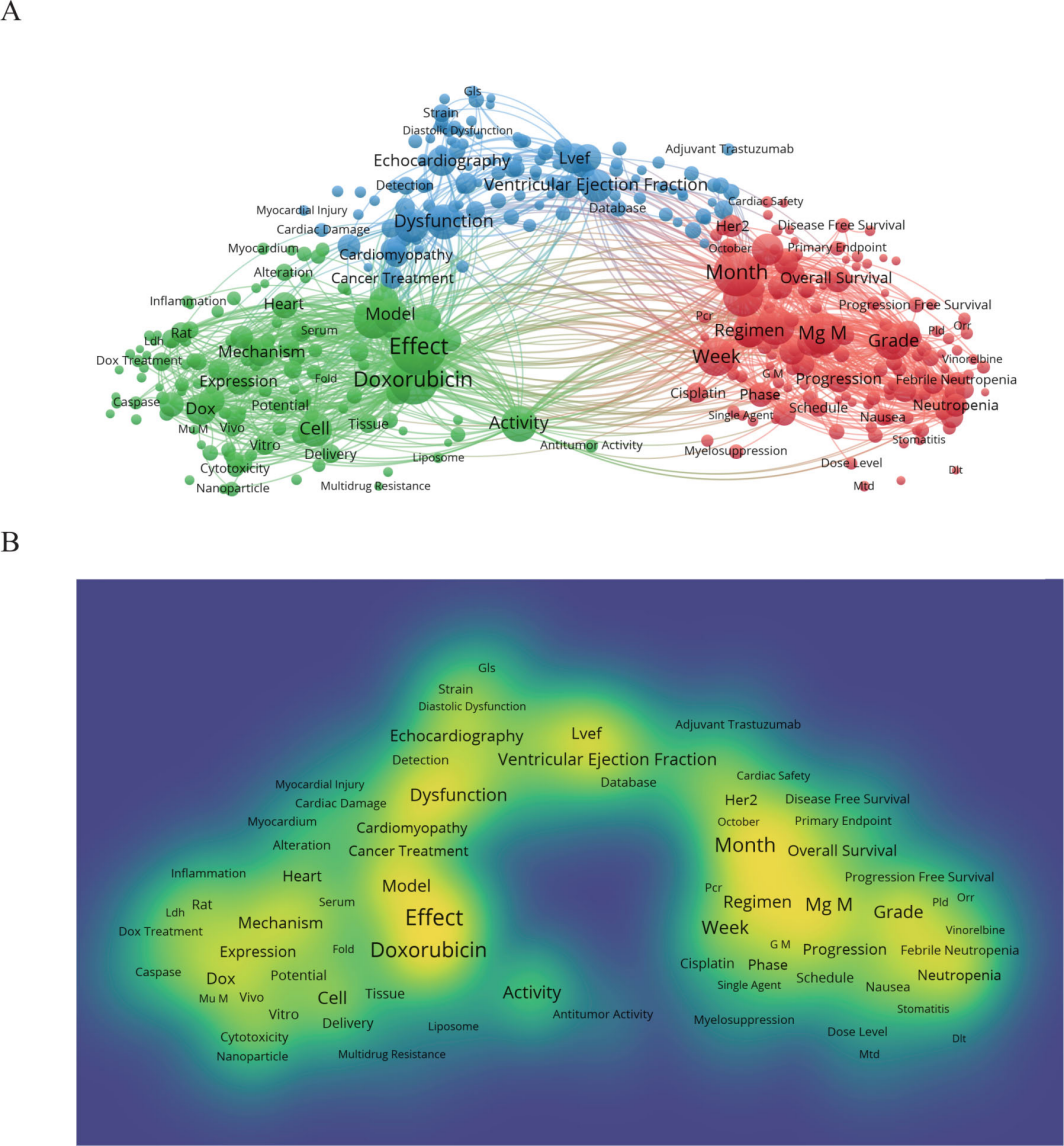
**(A)** illustrates the thematic network visualization, while **(B)** presents the density visualization. This chart selects the top 60% of theme words with a frequency greater than 40 words. These keywords can be roughly divided into three clusters, each representing a different meaning. Cluster One is related to time, Cluster Two is related to therapeutic drugs, and Cluster Three is related to therapeutic effects. The larger the circle, the more times the theme word appears **(A)**. Similarly, a darker color corresponds to a higher frequency of occurrence. **(B)**.

A total of 72,792 subject terms were extracted from 4,460 articles. Among these, 691 subject terms appeared more than 40 times. **Figure 4** features the top 60% (415 terms) selected to generate network and density visualizations (**Figure 4**). In **Figure 4A**, there are three main clusters comprising 415 nodes connected by 57,557 lines. These clusters are primarily dominated by the terms “Effect” “Dysfunction” and “Time”.

Network and density visualizations (**Figure 4B**) reveal that the three most frequent terms are “Effect” “Doxorubicin” and “Month” each appearing more than 1000 times, indicating that “Effect” is the most significant subject term. Following these top three, there are terms such as “Mg M” “Trial” “Level” “Cycle” “Week” “Grade” and “Activity”. The principle subject terms are closely associated with the actions and activities related to cancer treatment; hence, these articles are intrinsically linked to the mechanisms of tumor therapy. Of course, as treatment efficacy is addressed, various animal experiments are also mentioned, which is why terms like “Trial,” “Rat,” and similar ones appear.

## Discussion

### General information

In this study, we systematically searched the WOS database for literature on cardiotoxicity of chemotherapy published between 1994 and 2024. A bibliometric visualization analysis was performed on 4460 articles published in 941 journals. Since 1994, the number of related literature published has been increasing year by year. By examining the literature from 1994 to 2024, we have identified a significant growth in the number of publications, reflecting an increased focus on this critical issue within the oncology community.

Among these, the United States, China, and Italy are the countries with the highest number of publications in this field, contributing 1391 articles (31.19% of the total), 686 articles (15.38%), and 544 articles (12.20%), respectively. The United States and the United Kingdom have shown the most outstanding performance in international cooperation, especially the Dana Farber Cancer Institute in the United States (affiliated with Harvard Medical School), which has strong cooperation capabilities. The cooperation between research institutions in various countries and the United States helps to promote the development of this field.

In terms of journals, the Journal of Clinical Oncology not only publishes the most research articles in this field but also ranks first in co-citation frequency, demonstrating its importance in the research of cancer chemotherapy cardiotoxicity. It is worth noting that in the top ten most cited journals, all journals have a JCR partition of Q1, indicating their high influence in the field. However, despite China’s significant contribution in this field, none of the top ten journals have publishers from Asia, indicating the importance of establishing internationally influential journals in Asia for the development of this field.

Co-citation, which refers to references cited together in another publication, was detected using CiteSpace in this study. The greater the number of times an article is cited, the more significant it becomes within a specific field. The first article holds the highest number of citations, totaling 136, ranking it first. This article primarily focuses on cancer treatment and cardiovascular toxicity, encompassing cardiovascular complications in cancer treatment, strategies for preventing and mitigating cardiovascular complications in cancer treatment, and long-term monitoring plans for cancer survivors. The second article delves into the types of cardiotoxicity caused by anthracycline drugs and prospectively assesses the incidence, timing, and clinical relevance of cardiotoxicity (21). The third article aims to point out that cardiac dysfunction is a serious side effect caused by certain cancer-targeted treatments, which may hinder the effective implementation of treatment, damage the quality of life of patients, and even have adverse effects on the actual survival of cancer patients. It is designed to describe guidelines for preventing and detecting cardiac dysfunction in adult cancer survivors (26). The American Society of Echocardiography and the European Society for Cardiovascular Imaging evaluate patients undergoing cancer treatment, providing a comprehensive overview of the definition, classification, and mechanisms of toxicity. Additionally, they employ echocardiography to assess and detect cardiac function in cancer patients (27). In summary, these high-citation articles present the latest progress in the study of the effects of chemotherapy on cardiac toxicity, providing us with a deeper understanding of the continuously evolving knowledge system in this field.

### Knowledge base

The significant progress made in the field of cancer treatment has led to an increasing number of cancer survivors year by year, resulting in a growing number of cancer survivors facing various long-term complications during and after cancer treatment. In most cases, the deaths of cancer survivors are due to cardiac toxicity caused by cancer treatment (CTIC) (28). Cardiotoxicity refers to heart damage and dysfunction, which is the main reason for the high incidence of complications in cancer treatment and one of the biggest challenges in the overall management of cancer survivors (29). Studies have shown that cancer patients are more likely to experience serious cardiovascular events such as heart failure, myocardial infarction, arrhythmia, and heart valve disease compared to age - and gender-matched control groups (30). CTIC is not only associated with many targeted therapies and immunotherapies but also closely related to anthracycline chemotherapy drugs (29, 31). Many studies have shown that the occurrence of cardiotoxicity of anthracycline chemotherapy is closely related to some factors, such as patient’s age and sex, smoking, combined cardiovascular risk factors such as hypertension, diabetes, dyslipidemia, obesity, history of mediastinal or cardiac irradiation, anthracycline dosage form, cumulative dose, mode of administration, combined drug use, genetic background, etc (32, 33). At present, controlling traditional cardiovascular risk factors, strengthening medical history assessment, implementing exercise therapy, and using cardioprotective drugs are considered the main preventive measures (34–38). The European Society of Cardiology (ESC) Council on Cardio-Oncology aims to improve the prevention, diagnosis, and management of cardiovascular diseases (CVD) caused by cancer therapies. This includes addressing complications such as heart failure, arrhythmias, and coronary artery disease resulting from chemotherapy, radiotherapy, or immunotherapy. In 2022, the ESC released guidelines for cardiac oncology (39) defining and grading asymptomatic cancer therapies related cardiac dysfunctions: ① Mild LVEF ≥ 50% and GLS decreased by more than 15% compared to baseline, with/without newly developed elevated cardiac markers (cTnI/cTnT>99th percentile, BNP ≥ 35pg/mL, NT proBNP ≥ 125pg/mL or significantly elevated compared to baseline); ② Moderately, newly diagnosed LVEF with a decrease of more than 10% from baseline and LVEF of 4049%, or newly diagnosed IVEF with a decrease of no more than 10% from baseline and LVEF of 40-49%, accompanied by a decrease of more than 15% in GLS from baseline or an increase in one of the newly diagnosed cardiac markers; ③ The severity of newly diagnosed IVEF has decreased to below 40%. At the same time, CTRCD with symptoms (heart failure) was also classified as follows: ① Mild: mild symptoms, no need for intensified treatment; ② Moderate: Outpatient intensive diuretic and anti-heart failure treatment is required; ③ Severe: hospitalization is required. ④ Extremely severe: cardiac stimulants, circulatory support, or heart transplantation are needed. A comprehensive understanding of the cardiotoxic mechanisms of anticancer drugs is crucial for optimizing therapeutic strategies and minimizing adverse cardiovascular events. Current evidence elucidates the heterogeneous nature of these mechanisms (39): ①Anthracyclines exert their cardiotoxic effects through the generation of oxygen free radicals, which induce oxidative stress and subsequent damage to cardiomyocytes. This damage manifests in a dose-dependent manner, with cumulative exposure exacerbating myocardial impairment. ②HER2-targeted therapies, while effective in oncology, may compromise myocardial repair mechanisms. Their concomitant use with anthracyclines amplifies the synergistic cardiotoxicity risk, necessitating rigorous monitoring and risk-benefit assessment. ③Immune checkpoint inhibitors have emerged as a distinct class of cardiotoxic agents. By dysregulating immune homeostasis, these agents trigger excessive inflammatory responses targeting the myocardium, precipitating myocarditis and other immune-related cardiac complications. ④VEGF inhibitors impair angiogenesis, leading to hypertension and cardiac dysfunction. The anti-angiogenic effects disrupt physiological vascular homeostasis, contributing to elevated blood pressure and reduced cardiac performance. ⑤Additional agents, including tyrosine kinase inhibitors (TKIs) and alkylating agents, exert cardiotoxic effects via diverse mechanisms. TKIs may induce QT prolongation and heart failure, whereas alkylating agents can cause direct myocardial injury and pericardial disease. At present, it is still difficult to accurately detect which patients are at risk of developing CTIC early in clinical practice. Even if early detection of CTIC can quickly stop cancer treatment for affected patients to minimize further cardiac damage, it will affect the outcome of cancer treatment. Developing assisted cardioprotective therapies may completely change the outcomes of cancer survivors, meaning that effective cancer therapies can not only continue to be used but also effectively prevent the occurrence of cardiotoxicity. Therefore, there is an urgent need to identify new therapeutic agents that can prevent and/or reverse cardiovascular adverse reactions caused by cancer treatment without compromising their anti-cancer therapeutic benefits. Basic research has shown that chemotherapy drugs can cause an increase in low frequency (LF) and a decrease in high frequency (HF) and LF/HF in rats, leading to autonomic nervous system dysfunction (40). However, there is still a lack of basic experimental research to clarify the pathophysiological mechanisms, which affects the establishment of early diagnostic indicators for various chemotherapy drugs such as doxorubicin induced cardiac toxicity.

### Hotspots and frontiers

Keyword co-occurrence analysis plays a pivotal role in bibliometric studies, while data visualization serves to illustrate the focal points within a research domain (41). In summary, over the past 30 years, in the field of chemotherapy-induced cardiotoxicity in cancer patients, the keywords that have received the most attention include breast cancer, doxorubicin, trial, adjuvant chemotherapy, paclitaxel, risk, cyclophosphamide, oxidative stress, and combination. These keywords represent the hotspots in this research field during these years. Anthracyclines, alkylating agents, paclitaxel, and fluorouracil are common chemotherapy drugs associated with cardiac toxicity. Doxorubicin, a quintessential anthracycline drug, exhibits the most characteristic cardiotoxicity among all anthracycline agents, which is commonly utilized in the treatment and palliative care of breast cancer, lymphoma, leukemia, sarcoma, and certain gastrointestinal malignancies (22, 42). Anthracycline-induced myocardial damage is caused by a variety of factors, including reactive oxygen species production, mitochondrial damage, calcium overload, and apoptosis. Oxidative stress is one of the recognized mechanisms (43). Paclitaxel, a taxane classed as a mitotic spindle poison, exhibits an associated frequency of cardiac complications of 29%, with approximately 75% of these cases being asymptomatic bradycardia (44–46). The clinical manifestations of these symptoms typically occur within several hours and are reversible upon cessation of treatment (45). Furthermore, studies have indicated that the concomitant use of paclitaxel with anthracycline-based chemotherapy may potentiate the cardiotoxic risks associated with anthracycline agents (47). Cyclophosphamide, an alkylating agent with antitumor, immunosuppressive, and immunomodulatory effects, can also elicit cardiotoxicity, especially at high doses (48, 49). The cardiotoxicity associated with cyclophosphamide may manifest as arrhythmias, heart failure, hypotension, hemorrhagic myocarditis, and pericardial pathology (50). Oxidative stress and direct endothelial capillary damage are considered to be the mechanisms underlying its cardiotoxic effects (50). Fluoropyrimidine drugs, including 5-fluorouracil and capecitabine, are commonly utilized in the treatment of malignancies of the colorectal, breast, gastrointestinal, and head and neck regions. The mechanism underlying the cardiotoxicity induced by fluoropyrimidines remains elusive; however, the depletion of nitric oxide is posited as the most prevalent etiology (51). Since the 1960s, combination chemotherapy, which involves the concurrent administration of two or more chemotherapeutic agents, has emerged as a pivotal strategy for treating various cancers. Over the decades, this approach has given rise to a multitude of regimens, including paclitaxel (PTX)-based combinations (52), and anthracycline-based combinations, among others. Compared to monotherapy, combination chemotherapy enhances the sensitivity of cancer cells to drugs, modulates diverse signaling pathways within malignant cells, and reduces the dosage of individual drugs to mitigate side effects (53, 54). However, the concomitant use of multiple agents also heightens the risk of toxicity in patients (42). In addition, single chemotherapy may induce multiple drug resistance (55).

The emergence of ‘children’ as the largest cluster (Cluster 0) in the keyword clustering analysis indicates that chemotherapy-related cardiotoxicity in pediatric populations has attracted substantial attention. Children exhibit differences from adults in drug metabolism, including absorption, metabolism, and excretion of medications. Concurrently, studies have demonstrated that tissues in children are more sensitive to apoptotic signals, and the developmental regulation of mitochondrial apoptosis by c-Myc may be a reason why pediatric cancer patients are more susceptible to chemotherapy toxicity than adult cancer patients (56). For decades, anthracycline-based chemotherapy regimens have been extensively utilized in the treatment of pediatric cancer patients. In contemporary protocols, it is estimated that over half of the pediatric cancer patients receiving treatment are administered some form of anthracycline therapy (57). The majority of pediatric cancer survivors live for at least 10 years after successful cancer treatment (58). How to mitigate cardiotoxicity through primary prevention and effectively managing toxicity that has already occurred to minimize the ongoing incidence is crucial for improving the chronic health status and health-related quality of life of pediatric malignancy survivors.

Keyword burst analysis can showcase the frequently cited keywords in specific field articles during a certain period. It can indicate newly emerging hotspots in the research field. This research provides the top 50 most cited keywords, which may indicate the current hot topics in the field of cardiac toxicity induced by cancer chemotherapy. The graph shows that keywords such as echocardiography, nanoparticles, mechanisms, prevention, society, oxidative stress, inflammation, consensus, and global longitudinal strain have recently emerged and may continue to receive attention. The emerging keywords suggest that the current research frontier focuses on exploring the mechanisms of chemotherapy-induced cardiac toxicity (59, 60), monitoring and assessment of chemotherapy-induced cardiac toxicity (61–64), as well as prevention, reduction, and treatment of chemotherapy-related cardiac toxicity (65–69). Echocardiography is considered the standard for cardiac imaging assessment in patients during and after cancer treatment (69). Left ventricular ejection fraction (LVEF) is the most commonly used and comprehensive parameter for heart failure diagnosis, characterization, prognosis, monitoring, treatment decisions, and clinical trial eligibility (70). However, it lacks sensitivity in detecting subclinical changes in cardiac function induced by cardiotoxic therapies (71). The reduction in global longitudinal strain (GLS) appears to be the most sensitive parameter for predicting early cardiac toxicity, with established diagnostic and prognostic value (71). In an international, multicenter, prospective, randomized controlled trial, patients guided by GLS showed a significant decrease in LVEF at 1-year follow-up when compared to those receiving cardiac protective therapy (CPT). Furthermore, GLS-guided CPT significantly reduced the substantial decline of LVEF into the abnormal range. These results support the application of GLS in monitoring cancer treatment-related cardiac dysfunction (CTRCD) (61). A retrospective cohort study highlighted GLS as a strong predictor of clinical events and future deterioration in LVEF in patients with heart failure with preserved ejection fraction (HFpEF) (72). Furthermore, research has indicated that the development of diastolic dysfunction serves as a robust predictive marker for subsequent anthracycline-induced cardiotoxicity (73). The assessment of diastolic function, in conjunction with other echocardiographic parameters, aids in the evaluation of the risk of anthracycline cardiotoxicity (73). The adverse effects of chemotherapy drugs are contingent upon the dosage and duration of their administration. Cardiac tissue is one of the primary targets of chemotherapy drugs, and long-term and high-concentration use of these agents can lead to cardiotoxicity.

Consequently, two strategies can be employed to address this issue: reducing the level of drug administration and combining it with protective agents. Typical nanomaterials possess several common characteristics: a high surface-area-to-volume ratio, enhanced electrical conductivity, superparamagnetic behavior, spectral shifts in light absorption, and unique fluorescent properties (74). These attributes enable the application of nanomaterials in drug delivery and controlled release (74). Targeted delivery, based on nanomaterials, is one of the main advantages of cancer treatment over free drugs (74). **It** helps to reduce toxicity in normal cells, minimize side effects, protect drugs from degradation, and enhance half-life, loading capacity, and solubility, thereby improving therapeutic efficacy (75–77). By facilitating targeted delivery and reducing accumulation in the heart, nanomaterials can mitigate cardiotoxicity (78).

### Research agenda for future work

With the continuous advancement of research on the cardiac toxicity induced by cancer chemotherapy, an increasing number of relevant articles are being published in major journals, indicating a growing focus in this field. The United States, China, and Italy are at the forefront of research in this area. The United States and the United Kingdom demonstrate the strongest collaborative efforts compared to other countries. The collaborative capabilities of the Dana Farber Cancer Institute at Harvard Medical School are particularly robust, and institutions worldwide could enhance their cooperation with U.S. counterparts to further the development of this field.

Current research frontiers involve exploring the mechanisms of chemotherapy-induced cardiac toxicity, monitoring and evaluating such toxicity, preventing, reducing, and treating chemotherapy-induced cardiac toxicity. The mechanisms of cardiotoxicity associated with antitumor therapy are complex and diverse, yet no studies have compared these mechanisms to date. It remains to be investigated which mechanism contributes more significantly to cardiotoxicity among different therapeutic approaches. In particular, the use of nanodelivery systems to enhance drug bioavailability, reduce accumulation in the heart, minimize side effects, and improve therapeutic outcomes through targeted delivery has emerged as a major research focus in recent years. Various types of nanoplatforms have been developed to mitigate cardiac toxicity; however, only a relatively small number of nanomedicines have been thoroughly developed and successfully integrated into clinical practice. This suggests that further exploration of the potential of traditional Chinese medicine to ameliorate cardiotoxicity induced by chemotherapy is highly promising.

Monoclonal antibodies (mAbs) have demonstrated remarkable therapeutic efficacy in tumor immunotherapy; however, their potential to induce cardiotoxicity warrants careful consideration. Notably, mAbs with distinct mechanisms of action exhibit significant disparities in their cardiotoxic profiles. mAbs directed against HER-2, such as trastuzumab, have been implicated in the impairment of cardiac function through interference with the survival signaling pathways in myocardial cells. Furthermore, the incidence of cardiotoxicity is significantly elevated when these mAbs are administered in combination with other therapeutic agents (79).Immune checkpoint inhibitors (ICIs), such as cytotoxic T-lymphocyte-associated protein 4 (CTLA-4) and programmed cell death protein 1/programmed death-ligand 1 (PD-1/PD-L1) monoclonal antibodies, function by blocking immune checkpoint molecules like CTLA-4 and PD-1/PD-L1. The disruption of inhibitory signals from tumor cells to T cells may concomitantly alleviate the suppression of T cells by myocardial cells. This dual relief of inhibition can precipitate hyperactivation of T cells within cardiac tissue, instigating autoimmune responses and subsequent myocardial injury. Notably, this risk of cardiotoxicity is significantly amplified when ICIs are administered in combination with other therapeutic modalities (80).Emerging therapeutic mAbs, such as those targeting interleukin-1β (IL-1β), have exhibited promising cardioprotective properties. These mAbs function by inhibiting inflammatory pathways and fibrotic processes, which in turn contributes to the improvement of cardiac function (81).

Certain traditional Chinese medicinal practices have been identified as beneficial in mitigating cardiotoxicity induced by chemotherapy. Animal studies have demonstrated that electroacupuncture at the PC6 point can prevent doxorubicin-induced cardiotoxicity by reducing the generation of nitric oxide induced by doxorubicin (82). Another animal study has indicated that dandelion may exert its cardioprotective effects in doxorubicin-treated mice through the modulation of P-glycoprotein (83). Additionally, Shenlijia has been shown to mediate the upregulation of cardioprotective genes in the hearts of rats with doxorubicin-induced chronic heart failure (84). Furthermore, the silkworm silk peach can significantly reduce the levels of lipid peroxidation (LP) and cardiac injury marker enzymes, increase the levels of glutathione (GSH) and superoxide dismutase (SOD), reverse electrocardiogram changes, and prevent the reduction of heart weight (85). The modified gardenia liver plant decoction (ZGCT) administered to a patient with refractory acute lymphoblastic leukemia for two months improved the patient’s cardiac edema (86). Pretreatment with the aqueous extract of goji berries significantly prevented the loss of myofibrils in doxorubicin-treated rats and improved arrhythmias and conduction abnormalities (87).

Currently, cardiology has evolved into a new subspecialty of cardiology. Traditionally centered on drug toxicity, an increasing recognition of potential shared underlying causes between cancer and heart disease has led to an expansion of the field of cardioncology (88). Smoking, obesity, hyperlipidemia, and diabetes are significant risk factors for cardiovascular diseases, and individuals with these conditions may also be predisposed to cancer (88). Furthermore, the potential link between existing cardiovascular diseases and subsequent malignancies, known as reverse cardioncology, deserves attention. Patients with cardiovascular diseases are at a higher risk of developing cancer compared to the general population. Studies indicate that patients with heart failure (HF) have a 70% higher risk of developing cancer than those without HF, and this cancer risk increases over time (89). Moreover, in the absence of heart failure, early cardiac remodeling can promote tumor growth. Research by Abraham et al. (90)found that aortic constriction can facilitate the metastasis of two types of cancer cells: the PyMT (polyoma middle T) breast cancer model and the syngeneic Lewis lung cancer model. Similarly, drug-induced cardiac hypertrophy (91) [309] has been demonstrated to accelerate tumor growth and lead to the colonization of metastatic tumors through alterations in secreted proteins.

Therefore, there is a need to strengthen preclinical and translational research in the future. Additionally, enhancing collaboration and communication among experts in cardiology, oncology, pharmacy, laboratory medicine, and related fields is crucial to further expand and refine the assessment, monitoring, prevention, and treatment measures for chemotherapy-induced cardiac toxicity in cancer treatment, ultimately reducing side effects and improving patient outcomes.

### Limitations

This study pioneers the quantitative analysis and visual representation of literature on cardiac toxicity in cancer chemotherapy by employing co-occurrence and citation analysis techniques from VOSviewer and CiteSpace, showcasing a degree of originality. However, it is essential to recognize its limitations. Initially, the investigation solely extracted data from the Web of Science (WOS) database, potentially overlooking journal articles not indexed in WOS. Secondly, it focused exclusively on academic papers, neglecting other forms of publications like editorials and books. Moreover, the study only encompasses articles from the SCIE database starting from its inception in 1994 up to the publication cutoff date of January 21, 2024. As the Web of Science (WOS) database is regularly updated, some 2024 literature might have been omitted, possibly affecting the completeness of our research findings concerning the periods before 1994 and in 2024. Lastly, the study only includes English-language research, potentially missing valuable literature in other languages. Nonetheless, considering the broad coverage and credibility of WOS, conclusions drawn solely from WOS database analysis maintain a certain level of reliability.

## Summary

In conclusion, we conducted a visual analysis of cardiac toxicity induced by cancer chemotherapy using various bibliometric software tools. Our study aimed to delineate the publication characteristics in this specific domain, scrutinize the most influential countries, institutions, journals, and authors, and elucidate the research hotspots and frontiers within this realm. Since 1994, research pertaining to cardiac toxicity induced by cancer chemotherapy has exhibited a consistent upward trajectory, notwithstanding fluctuations in publication volume. Noteworthy contributions to this domain have emanated from 100 countries/regions and 4343 research institutions. The United States, China, and Italy have emerged as leading contributors in terms of the sheer number of published articles. The Journal of Clinical Oncology stands out as the most prolific journal in this sphere and also boasts the highest citation frequency. Notably, author Bonnie Ky has emerged as prolific contributors within this field. The prevalent keywords encompass “breast cancer,” “doxorubicin,” “heart failure,” “trial,” “adjuvant chemotherapy,” “paclitaxel,” “risk,” “echocardiography,” “nanoparticles,” “mechanisms,” “prevention,” “society,” “oxidative stress,” “inflammation,” “consensus,” and “global longitudinal strain,” encapsulating the cutting-edge research frontiers within this domain. Presently, the research focal points in this area predominantly revolve around elucidating the mechanisms underlying chemotherapy-induced cardiac toxicity, monitoring and assessing such toxicity, and devising strategies for its prevention and management.

## Data Availability

The original contributions presented in the study are included in the article/supplementary material. Further inquiries can be directed to the corresponding author.
